# Aggressive Thyroid Carcinomas Clinical and Molecular Features: A Systematic Review

**DOI:** 10.3390/ijms26125535

**Published:** 2025-06-10

**Authors:** Sorina Schipor, Mihai Alin Publik, Dana Manda, Mihail Ceausu

**Affiliations:** 1 Department of Research, “C.I. Parhon” National Institute of Endocrinology, 011863 Bucharest, Romania; sorina.schipor@parhon.ro; 2 Department of Pathology, “C.I. Parhon” National Institute of Endocrinology, 011863 Bucharest, Romania; mihaipublik@gmail.com (M.A.P.); mihail.ceausu@umfcd.ro (M.C.); 3 Department of Pathology, “Carol Davila” University of Medicine and Pharmacy, 020021 Bucharest, Romania

**Keywords:** thyroid carcinoma, anaplastic, differentiated high grade, poorly differentiated, aggressive, DHGTC, PDTC, ATC

## Abstract

Aggressive thyroid carcinomas are rare malignancies characterized by a high impact on patient’s lives and poor prognosis. The available literature is scarce presenting divergent data concerning the clinical outcomes, prognostic factors and variable mutational signature studies. We aim to collect data from the literature and assemble a systematic review. The literature from 2007 until May 2025 was searched using PubMed. Studies bearing data concerning clinical aspects, prognostic outcomes, or molecular characteristics of differentiated high-grade (DHGTC), poorly differentiated (PDTC), and anaplastic thyroid carcinomas (ATC) were retrieved. Original articles in English, ethically conducted on human patients, were selected. From 688 articles, 39 were included. **DHGTC** has a good 5-year survival rate (5YSR) of 76%, 23.18% metastasis rate, 42.23%, lymph node involvement (LNI), 61.44% extrathyroidal extension (ETE), majority being diagnosed in stage III. **PDTC** has an intermediate 5YSR of 65.71%, 21.17% distant metastasis, 32.22% LNI, and 55.19% ETE, majority diagnosed in stage III. **ATC** has a grim 2-year survival rate of 11.15%, 42.15% metastasis, 44.14%, LNI, and 58.51% ETE, majority presented in stage IV-B. Mutational profiling shows that each carcinoma has its unique set of molecular alterations. Most positive prognostic comes for DHGTC, then PDTC, and finally, ATC.

## 1. Introduction

According to the Global Cancer Observatory, thyroid cancer is the seventh most common malignancy, spanning an incidence of 821,214 new cases annually. It is also the 24th cause of cancer-related mortality, accounting for a high number of 47,507 deaths each year. This results in a significant social burden and an estimated economic impact of over EUR 40.5 million annually, as reported in France [1,2].

The 2022 World Health Organisation (WHO) Classification of Tumors of Endocrine Organs (beta) categorizes tumors of follicular origin into two groups. The first group includes the differentiated thyroid carcinomas (DTC), encompassing follicular, papillary, oncocytic, and the invasive encapsulated follicular variant of papillary carcinoma [3,4]. The second group is composed of the less differentiated malignancies, namely anaplastic thyroid carcinoma (ATC), differentiated high-grade thyroid carcinoma (DHGTC), and poorly differentiated thyroid carcinoma (PDTC), all classified under the term high-grade follicular cell-derived non-anaplastic thyroid carcinoma [3,5]. The differentiated group accounts for approximately 90% of all thyroid cancers, while the less differentiated types represent only 10% [5,6,7,8].

This review focuses on the less differentiated category of thyroid malignancies, that express a much more aggressive behavior. These malignancies are characterized by poor survival rates, high rates of distant metastasis at upon first presentation, frequent lymph node involvement, large tumor size, and reduced avidity for radioactive iodine, when compared to DTCs [9]. Treatment sometimes proves to be a significant challenge in such cases. DTCs cumulate excellent 5-year survival rates of over 95%. On the contrary, ATC has significantly grimmer outcomes, with 5-year survival rates ranging from 8.1% to 12%, and a poor median 1-year survival rate of only 20–40% [10,11,12,13]. Intermediate survival rates reflect the intermediately aggressive behavior of PDTC, with a 5-year survival rate ranging from 44% to 85% [14].

The stepwise accumulation of mutations ultimately leads to the disruption of normal signaling pathways, resulting in the dysregulation of the cell cycle, altered cellular function, and anarchic proliferation [15,16]. Histologically and phenotypically, these changes are reflected in malignant behavior [15,17,18]. Molecular analysis through DNA sequencing has provided extensive expression data and prognostic value for various genes implicated in neoplasia; however, studies often offer variable conclusions and results [4,19,20,21].

Furthermore, many independent studies did not manage to cumulate a satisfactory number of patients alone. Widely varying results are presented in the literature, underlining the need for quantitative standardization, both in clinicopathological and mutational profiling of thyroid cancers. This review aims to quantitatively evaluate the current data concerning clinical and prognostic factors for aggressive thyroid carcinomas, integrate these findings into the molecular landscape, and compare the three aggressive thyroid neoplasms.

Objectives: To review current data on the clinicopathological characterization of aggressive thyroid carcinomas, including variables such as metastasis rates upon initial presentation, extrathyroidal extension, lymph node involvement, mean tumor size, mean age at presentation, TNM stage, and survival. Additionally, we aim to examine the mutational profile associated with these malignancies.

## 2. Method

This report follows Preferred Reporting Items for Systematic Reviews and Meta-Analysis (PRISMA) Statement 2022 [22]. A systematic literature search was conducted regarding the clinicopathological characterization of patients with DHGTC, PDTC, and ATC. A parallel search was performed for data on the mutational profiles of the same cancers, utilizing the PubMed Library.

### 2.1. Eligibility Criteria

We included original articles written in English that focused on ATC, PDTC, or DHGTC, appropriately conducted on adult human patients, ethically approved, and providing clinical and/or mutational profile data. For mutational assessment, only studies employing Sanger sequencing, next-generation sequencing (NGS), or fluorescence in situ hybridization (FISH) were considered. Articles on PDTC and ATC were retrieved from 2007 onwards, following the Turin criteria, while those on DHGTC were selected from 2022, following its reclassification.

Pediatric population-based studies, reviews, meta-analyses, case reports, letters, expert opinions, and studies that did not present the number of patients involved were excluded.

Additionally, articles that primarily used immunohistochemistry for mutation assessment, as well as those based on cytology or lacking histological confirmation of the cancer type, were excluded from the analysis.

### 2.2. Search Strategy

We searched PubMed Database for articles published after 2007, using the formula (Poorly Differentiated [Title/Abstract]) OR Anaplastic [Title/Abstract] OR (Differentiated High Grade [Title/Abstract]) AND (Thyroid [Title/Abstract]) AND (Carcinoma [Title/Abstract]) AND ((Prognostic [Title/Abstract] OR Mutations [Title/Abstract])OR (Clinical [Title/Abstract])) NOT (Review [Publication Type] OR Meta-Analysis [Publication Type]), limited only to title and abstract.

Reviews and meta-analyses were excluded by adding restrictions in the query. The database was last searched on 3 May 2025. In addition, after eligible articles were selected, references were also manually searched for other relevant materials addressing our topic.

### 2.3. Selection Process

Three researchers (MC, DM, SS) equally split the total number of articles and independently reviewed the titles and abstracts. Consensus upon inconsistencies was established by discussion. In a last step, MC, SS, MAP independently screened the full text of the remained studies and reached a decision on inclusion or exclusion.

### 2.4. Data Extraction

Data were extracted manually by MC, DM and cross-checked by SS and MP. Any inconsistency was resolved by another full-text examination by another full-text analysis. Ultimately, aggregated data was loaded under tabular form in Microsoft Excel^®^ software, Microsoft Corporation, Redmond, WA, USA.

We collected data concerning clinicopathological aspects: metastasis rates upon initial presentation, extrathyroidal extension, lymph node involvement, mean tumor size, mean age at presentation, TNM stage, and survival, as well as genetic profiling: BRAF_V600E_, all RAS, TERT, TP53, PTEN, and PIK3CA. If the study did not report all of the aforementioned parameters, we only recorded the available data and only used those in the subsequent statistics.

### 2.5. Risk of Bias Assessment

Two reviewers MAP and MC independently assessed the risk of bias of each article by employing the JBI Critical Appraisal Checklist for Studies Reporting Prevalence Data Tool. Results can be seen in Supplemental File S1. Each question answered with yes was attributed 1 point. The sum was computed for every study. Studies cumulating over 5 points were considered of good quality, whereas studies over 4 were considered satisfactory.

### 2.6. Synthesis Method

Given the heterogeneity of the collected aspects, we opted for graphical representation as plots along with narrative synthesis. For each parameter we plotted the mean reported by each study, and computed the weighted average based on the number of patients included in each study. JASP Version 0.19.3, JASP Team (2024) was used for statistical analysis. A weighted three-way analysis of variance (ANOVA) was used for each parameter to assess the statistical significance of the differences across the three cancers. Weight was applied to account for individual study number of patients. Statistical significance was set at *p* < 0.05. Tukey’s post hoc test was performed. To ensure the validity of ANOVA results, we also performed Levene’s test for homogeneity of variances. Additionally, we provided the number of patients taken into analysis for every plotted parameter for transparency. All graphs were achieved by using GraphPad Prism version 8.0.2 for Windows, GraphPad Software, Boston, MA, USA. Confidence intervals of 95% were computed for every reported parameter (See Supplemental Table S1).

## 3. Results

**Figure 1:**
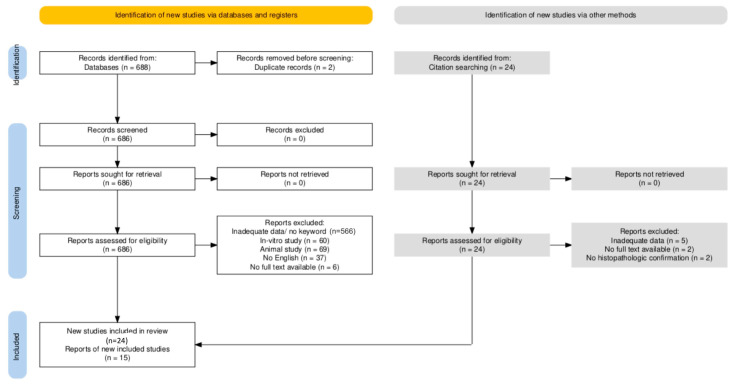
PRISMA flow chart showing the employed search strategy [59].

A total of 688 studies were identified for screening after the initial search on PubMed, and in a second step, another 15 were retrieved from references. In the last step, a total of 40 original articles meeting the aforementioned inclusion criteria were taken into account, as seen in Figure 1. In total, 29 studies (6588 patients) were included in the clinical assessment, whereas 19 (2085 patients) were included in the molecular section, some studies being used in both analyses if both kinds of data were provided. In the clinical section, we included, by type of cancer, 251 patients for DHGTC, 1985 for PDTC, and 4352 for ATC, whereas in the molecular assessment 468 DHGTC, 680 PDTC, and 937 for ATC. The main characteristics of each included study are summarized in Table 1 for the clinical part and Table 2 for the mutational profile section.

**Table 1:** Study characteristics for those included in the clinical section. DHGTC differentiated high-grade thyroid carcinoma; PDTC poorly differentiated thyroid carcinoma; ATC anaplastic thyroid carcinoma; DM distant metastasis upon presentation; ETE extrathyroidal extension; LNI lymph node involvement; MTS mean tumor size; SR survival rates.

Author, Year	Study Design	Patients	Median Age	Cancer Type	Parameters
Xu et al. 2022 [9]	Retrospective	164/200	55/59	DHGTC/PDTC	SR, ETE, LNI, DM
Panchangam et al. 2022 [14]	Retrospective	29	54	PDTC	SR, MTS, ETE, LNI, DM
Aslan et al. 2014 [23]	Retrospective	29	64.5	ATC	SR, MTS, ETE, DM
Brignardello et al. 2014 [24]	Retrospective	55	73.15	ATC	SR, ETE, DM
Duan et al. 2019 [25]	Retrospective	41/25	51/64	PDTC/ATC	MTS, ETE, LNI, DM
Evans et al. 2024 [26]	Retrospective Case–control	41	67.4	ATC	SR, DM
Fouchardiere et al. 2018 [27]	Retrospective	104	62	PDTC	SR, ETE, LNI, DM
Glaser et al. 2016 [28]	Retrospective	3552		ATC	SR, LNI, DM
Gu et al. 2024 [29]	Retrospective	15/42	52/64.5	PDTC/ATC	MTS, ETE, LNI, DM
Ibrahimpasic et al. 2014 [30]	Retrospective Case–control	91	59	PDTC	SR, ETE, LNI, DM
Jeong et al. 2023 [31]	Retrospective	14	47	DHGTC	MTS, ETE, LNI, DM
Jin et al. 2022 [32]	Retrospective	970		PDTC	LNI, DM
Kersting et al. 2021 [33]	Retrospective	51	58.5	PDTC	SR, ETE, DM
Kunte et al. 2022 [34]	Retrospective	19	60	PDTC	SR, LNI, DM
Landa et al. 2016 [35]	Retrospective	84/33	55/66	PDTC/ATC	DM
Patil et al. 2025 [36]	Retrospective	106	54	DHGTC/PDTC	SR, LNI, DM
Paunovic et al. 2016 [37]	Retrospective	150		ATC	SR
Saito et al. 2024 [38]	Retrospective	102	73	ATC	LNI
Sherman et al. 2011 [39]	Retrospective Case–control	75	68	ATC	SR, ETE, DM
Swaak-Kragten et al. 2011 [40]	Retrospective Case–control	37	63	ATC	SR
Thompson et al. 2023 [41]	Retrospective	17/24	64/58	DHGTC/PDTC	MTS, ETE, LNI, DM
Tondi Resta et al. 2024 [42]	Retrospective	32	52.6	DHGTC	MTS, ETE, LNI, DM
Wendler et al. 2016 [43]	Retrospective	100	70.5	ATC	MTS, LNI, DM
Wong et al. 2019 [44]	Retrospective	47	57	PDTC	MTS, ETE, LNI, DM
Wu et al. 2023 [45]	Retrospective	97	70	ATC	LNI, DM
Xu et al. 2023 [46]	Retrospective	210	60	PDTC	MTS, ETE, LNI
Yu et al. 2017 [47]	Retrospective	18	62	PDTC	SR, MTS, ETE, LNI, DM
Ravi et al. 2019 [48]	Retrospective	14	71.4	ATC	MTS, LNI, DM

**Table 2:** Study characteristics for those included in the mutational profile section. X shows the gene data that we could extract from each study. DHGTC differentiated high-grade thyroid carcinoma; PDTC poorly differentiated thyroid carcinoma; ATC anaplastic thyroid carcinoma; FISH fluorescent in situ hybridization; MSK-IMPACT Memorial Sloan Kettering-Integrated Mutation Profiling of Actionable Cancer Targets; NGS new generation sequencing; PCR polymerase chain reaction; RNA-seq RNA sequencing; WGS whole genome sequencing.

Author, Year	Type of Cancer	Patients	Yielded Parameters						Molecular Technique
			BRAF	RAS	TERT	TP53	PTEN	PIK3CA	
Bonhomme et al. 2017 [49]	ATC	94	x	x	x	x	x	x	NGS, FISH
Duan et al. 2019 [25]	PDTC	41	x	x	x	x	x	x	NGS
	ATC	25	x	x	x	x	x	x	
Fouchardière et al. 2018 [27]	PDTC	104	x	x	x				PCR
Gu et al. 2024 [29]	PDTC	9	x	x	x				Sanger Seq.
	ATC	24	x	x	x				
Landa et al. 2016 [35]	PDTC	84	x	x	x	x	x	x	NGS (Target Seq—MSK-IMPACT)
	ATC	33	x	x	x	x	x		
Latteyer et al. 2016 [50]	ATC	30	x	x		x			NGS
Pozdeyev et al. 2018 [51]	ATC	196	x	x	x	x			NGS (MSK-IMPACT)
Ravi et al. 2019 [48]	ATC	8	x	x	x	x	x	x	WES RNA-Seq
Saito et al. 2024 [38]	ATC	102	x	x	x	x			NGS (database)
Scholfield et al. 2025 [52]	DHGTC	252	x	x	x	x			NGS
Stenman et al. 2021 [53]	ATC	8	x		x	x	x	x	WGS RNA-Seq
Takano et al. 2007 [54]	ATC	20	x						Sanger seq
Tiedje et al. 2017 [55]	ATC	118	x	x	x	x		x	NGS
Toda et al. 2024 [56]	PDTC	104	x	x					NGS (database)
	ATC	130	x	x		x		x	
Torous et al. 2024 [57]	DHGTC	40			x				NGS
Wong et al. 2021 [44]	DHGTC	12	x	x	x	x			NGS
	ATC	33	x	x	x	x			
Xu et al. 2022 [9]	DHGTC	164	x	x	x	x	x	x	NGS (MSK-IMPACT)
	PDTC	87	x	x	x	x	x	x	
Xu et al. 2023 [46]	PDTC	87	x	x	x		x		
Yamazaki et al. 2024 [58]	PDTC	51	x	x	x	x	x	x	NGS (databse)
	ATC	110	x	x	x	x	x	x	

### 3.1. Clinical Outcome and Prognostic Considerations

**Figure 2:**
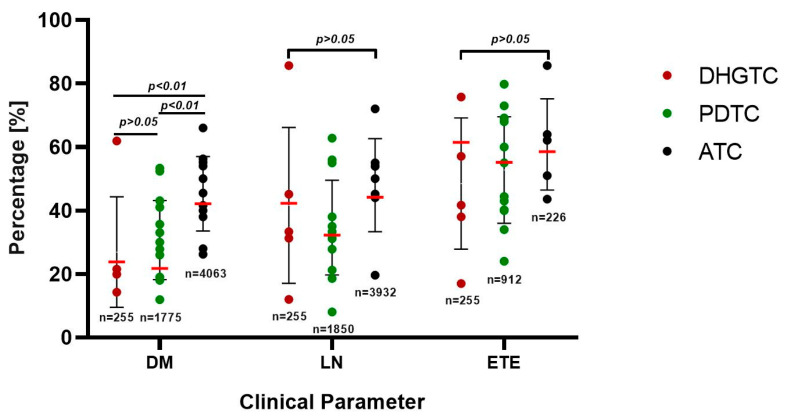
Metastasis rates upon initial presentation, lymph node involvement, and extrathyroidal extension values as percentages, represented for each type of cancer. On the vertical axes, we plotted points pertaining to the value provided by each study and the standard deviation. The red horizontal lines provide the weighted average for each parameter, weighted by the number of patients included in the studies. DM distant metastases; LN lymph node involvement; ETE: extrathyroidal extension; DHGTC: differentiated high-grade thyroid carcinoma; PDTC: poorly differentiated thyroid carcinoma; ATC: anaplastic thyroid carcinoma; n: number of patients included in each column.

a. **DHGTC** is a new subtype of thyroid carcinoma of follicular cell origin that has been included in the 2022 WHO Classification of Endocrine Tumors [42]. It is defined by high-grade features such as high mitotic activity, and tumor necrosis, but without anaplastic histotype, partially retaining some DTC markers [5]. This type of cancer has the best prognosis out of all three included subtypes. The weighted mean age at presentation was 54.8 years. As seen in Figure 2, DHGTC displays an overall weighted average of 23.81% metastasis rate upon initial presentation, 42.23%, lymph node involvement, and 61.44% extrathyroidal extension.

**Figure 3:**
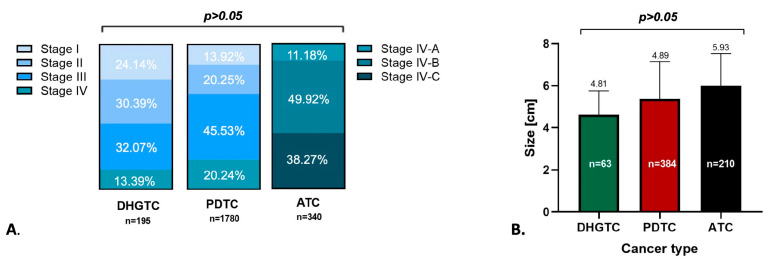
(**A**) AJCC Stage at presentation distribution by type of cancer, as weighted averages. (**B**) Weighted average of mean tumor size by cancer type, vertical bars plot for standard deviation. DHGTC: differentiated high grade thyroid carcinoma; PDTC: poorly differentiated thyroid carcinoma; ATC: anaplastic thyroid carcinoma, n: number of patients included.

**Figure 4:**
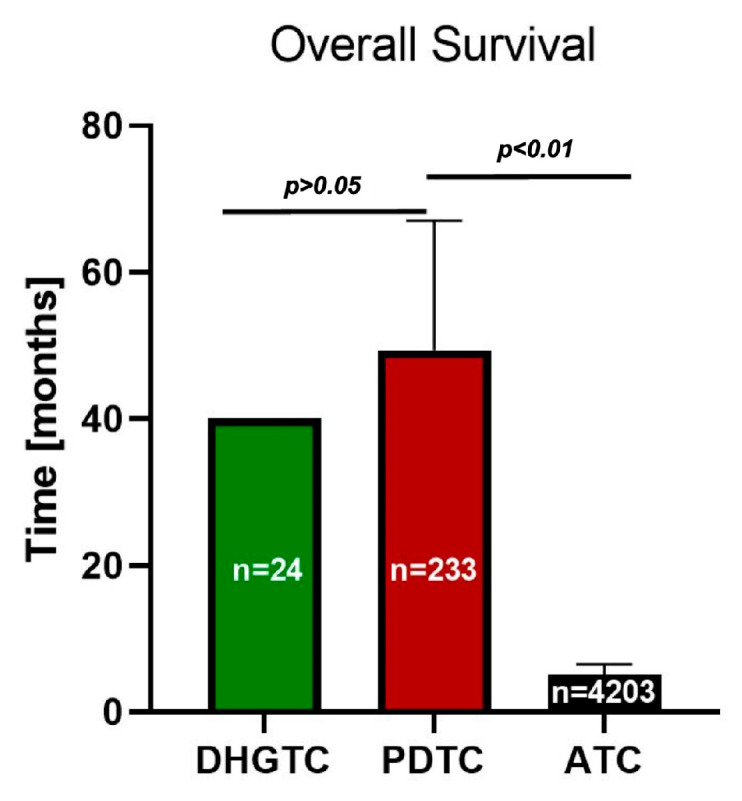
Overall survival for each type of cancer. Note the abruptly smaller overall survival in the case of ATC. DHGTC: differentiated high grade thyroid carcinoma. PDTC: poorly differentiated thyroid carcinoma; ATC: anaplastic thyroid carcinoma, n: number of patients included.

The weighted average tumor size was 4.81 cm. The majority of patients were diagnosed in AJCC stage III (31.77%), closely approaching that of stage II (30.18%), stage I (24.04%) and stage IV (13.30%) see Figure 3. This distribution generates a rather good 5-year survival rate of 76.00% (Figure 4). For 95% confidence intervals and weighted average for each parameter see Supplemental Table S1.

In general, this type of cancer does not exhibit radioactive iodine (RAI) avidity. Furthermore, two independent studies showed higher RAI avidity in PDTC compared to DHGTC [9,60]. Poor prognostic factors include tumor necrosis (Jeong et al. [31]), advanced age at diagnosis, and extrathyroidal extension (Jeong et al. [31] and Xu et al. [9]). Thompson et al. [41] quote similar metastasis rates upon presentation between DHGTC and PDTC, but the latter variant seems to have an increased rate afterward and in a shorter time frame. Interestingly, Ki67 does not seem to correlate with distant metastasis rates [41].

b. **PDTC** carcinoma is an aggressive clinicopathologic entity, showing behavior and outcome in between DTCs and ATC [13,61]. It is considered a rather rare cancer, with various incidence rates varying in between 2 and 15% of thyroid cancers [30]. The weighted mean age of patients was 58.14 years old. As seen in Figure 2, this malignancy is characterized by frequent 21.17% distant metastasis upon initial presentation, 32.22% lymph node involvement, and 55.19% extrathyroidal extension upon initial presentation. The weighted mean tumor size was 4.89 cm. The majority of patients presented late, AJCC Stage III (45.53%), with a modest distribution spanning stages I and II of only 13.92% and 20.15%, respectively, and 20.24% for stage IV (Figure 3). It exhibits a satisfactory overall survival (OS) of 55.8 months and a 5-year survival rate of 65.71% (Figure 4). Low RAI affinity further complicates the treatment and prognostic for this affliction, tumor size bigger than 4 cm being an excellent predictor for refractoriness as demonstrated by both La Fouchardiere [27] and Kersting [33].

Many studies incriminate different clinicopathological parameters as predictors for OS, such as advanced age at diagnosis, extrathyroidal extension, size > 4 cm, incomplete resection, and lack of encapsulation [9,13,27,44,62,63]. Xu et al. [46] demonstrates on a significant cohort that oncocytic hystotype results in worse overall outcomes. Despite a relatively small patient lot, Kunte et al. [34] underline that advanced T and N stages were bad prognostic factors, regardless of the therapeutic conduit. Their conclusion is in disagreement with Yu et al. [47], who regard surgery as a central piece for better outcome. Wong et al. [44] prove on a substantial cohort that tumor encapsulation and vascular invasion are strong prognostic factors for PDTC: the best 5-year disease-free survival being obtained for encapsulated tumor and focal vascular invasion (100%), 75% for extensive vascular involvement and a very poor 17% for widely extensive. See Supplemental Table S1 for 95% confidence intervals and weighted average of each clinical parameter.

c. **ATC** is by far the most aggressive and rapidly progressing form of thyroid neoplasia, accounting for approximately 1–4% of total thyroid cancer bourdain [5,64,65].

Histologically speaking, this tumor shows undifferentiated cells, high nuclear pleomorphism, atypical mitoses, and zones of DTC, or the patient may have a history of preceding DTC [5,29]. Many times, a pure histologic diagnosis and origin assessment may be challenging, in part because it shows no normal thyroid markers immunohistochemically [66]. The high number of gathered mutations makes the neoplastic cell almost entirely unresemblant of the normal follicular cell, also implying no avidity for RAI and thus limited therapeutic options [66]. ATC has the highest rate of metastasis upon presentation of 42.15% when compared to PDTC and DHGTC (*p* < 0.01), the highest lymph node involvement of 44.14%, and 58.51% extrathyroidal extension (Figure 2). The weighted average tumor size is approximately 5.93 cm.

The American Joint Committee for Cancer (AJCC) Cancer Staging Manual classifies ATC as stage IV from the start, so it is already a late-stage disease, and the majority of patients present in stage IV-B 49.92%, IV-A 11.81%, IV-C 38.27% (Figure 3). The prognostic is grim with an overall survival (OS) of just 3.7 months, and 6-month, 1-year, and 2-year survival rates of 51.05%, 19.06%, and 11.15%, respectively (Figure 4). The difference in OS between PDTC and ATC is strongly statistically significant: 57 months vs. 3.7 months, respectively (*p* < 0.001). See Supplemental Table S1 for statistical parameters.

The poor clinical prognostic factors cited in literature are: advanced age, metastases at presentation, and incomplete resection [23,28,48,66,67]. Wendler et al. [43] demonstrate on a consistent cohort that strong positive prognostic factors are radical surgery, chemotherapy, and external radiotherapy, whereas Sherman et al. [39] studied the same variables on a smaller group that did not manage to reach statistical significance.

On the other hand, numerous studies demonstrate that surgery achieving complete resection is a powerful positive prognostic factor [23,24,28]. Furthermore, Brignardello et al. [24] prove on a consistent lot of 55 patients that early complete resection uniformizes survival between stages IV-A, IV-B, and IV-C. This suggests that surgery may be a tool for improving survival and quality of life even for inoperable IV-C patients and should not be limited to the first two stages. Glaser et al. [28] assembled a cohort of 3552 patients, highlighting complete resection through total thyroidectomy, along with high-dose external radiotherapy as better OS predictors.

Targeted immunotherapies are promising future prospects, inducing importantly higher OS in patients who received this kind of therapy versus no targeted therapy, as proven by Evans et al. [26]. Interestingly, Ahn et al. [68] demonstrate on a lot of 35 patients decreased OS of 3 months in patients with ATC and low lymphocyte to monocyte ratio versus normal ratio with OS of 9.5 months.

### 3.2. Molecular Landscape

**Figure 5:**
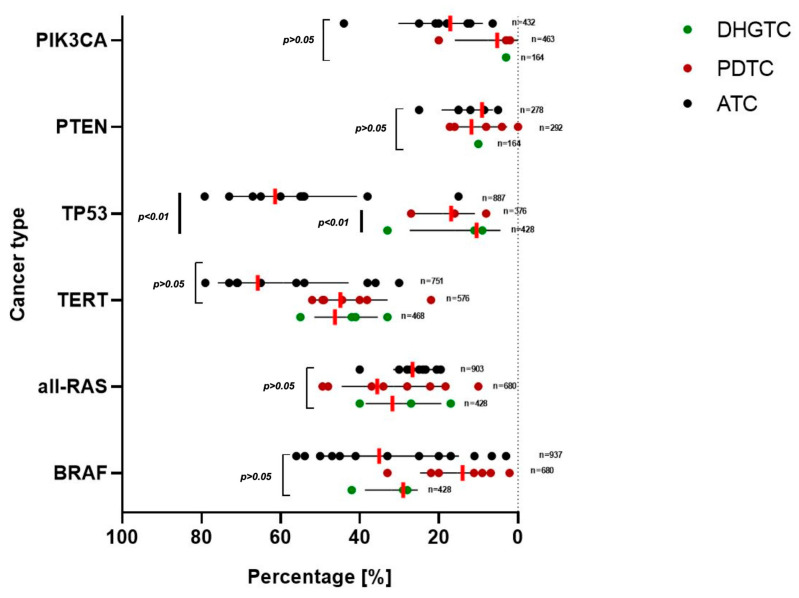
Mutation prevalence in percentage, represented for each type of cancer. On the horizontal axis, we plotted the value provided by each study and the standard deviation. The red vertical lines provide the weighted average for each parameter, weighted by the number of patients included in the studies. Only TP53 difference is statistically significant between DHGTC-PDTC and DHGTC-ATC (*p* < 0.01). DHGTC: differentiated high grade thyroid carcinoma; PDTC: poorly differentiated thyroid carcinoma; ATC: anaplastic thyroid carcinoma, n: number of patients included for each parameter.

Thyroid tumorigenesis is considered a multi-step process in which the cell clone gathers an increasing number of mutations, as proven by the fact that many dedifferentiated thyroid carcinomas coexist with zones of DTCs [29,69]. This means that the cell undergoes a series of epigenetic events that makes it progress from benign to malignant well differentiated, and ultimately to poorly differentiated or anaplastic histotype [15,70,71]. The more genetic aberrations the cell gathers, the more aggressive and poorly differentiated the cancer is [35,69,72]. By dividing mutations into early and late events, literature separates the initial drivers of neoplasia, such as BRAF and RAS, and late mutations such as TERT, TP53, PTEN, and PIK3CA rearrangements that account for dedifferentiation and aggressive behavior [17,18,72]. Every thyroid carcinoma seems to have its proprietary molecular signature made up of different mutations occurring in a particular manner.

a. **DHGTC** exerts equal prevalence in BRAF and RAS: 28.98% and 31.70%, respectively; 46.62% for TERT, 10.44% and 10.00% for TP53 and PTEN, respectively. Subsidiary, it has low prevalence rates of PIK3CA modifications of about 3%, explaining the lower aggressiveness of this malignancy (Figure 5). See Supplemental Table S2 for 95%CI and weighted average. As seen in the studies of Xu et al. [9] and Scholfield et al. [52], BRAFV600E and TERT mutations lower the distant metastasis survival rates, and BRAF is associated with invasive disease, while RAS is for angioinvasive behavior.

b. **PDTC** is a RAS and TERT-driven neoplasia, with high prevalence rates of 35.57% and 44.83%, respectively. The other studied mutations BRAF, TP53, PTEN, and PIK3CA have expressions of 13.96%, 16.90%, 11.76%, and 5.21%, respectively (Figure 5) (Supplemental Table S2). All genes seem to have slightly higher prevalences than in DHGTC, but interestingly, BRAFV600E has a lower value of less than half, observation in agreement with Xu et al. [9] who proved in a substantial cohort that RAS mutations associated with PDTC, whereas BRAF associates with DHGTC. Furthermore, Landa et al. [35] demonstrates that BRAFV600E mutation is mutually exclusive with RAS. Two studies showed that TERT and RAS could be linked to RAI refractoriness [27,58]. BRAF plus TERT coexisting mutations seem to predict a worse metastasis prognostic [9,69]. Duan et al. [25] demonstrates in a cohort of 41 patients that the combination of TERT and PIK3CA mutation is a predictor for bad overall prognostic.

c. **ATC** has the highest mutational burden [51,56]. BRAF and RAS mutations have a prevalence of 35% and 26%, and Stenman [53] and Landa [35] show that they are mutually exclusive. Late events, such as TERT and TP53 have very high rates (65% and 61%), meaning that ATC is driven by these late modifications (Figure 5). PTEN and PIK3CA have low prevalence rates of 9.05% and 17.09%, respectively (Supplemental Table S2). Statistically significant differences appeared in TP53 expression between DHGTC-PDTC and DHGTC-ATC, respectively (*p* < 0.01). BRAF mutation alongside TP53 mutation is a predictor for shorter OS, as shown by multiple studies (Toda [56], Gu [29]).

## 4. Discussion

Aggressive thyroid carcinomas still represent an important cause of mortality and morbidity, and therapeutic resources are very poor [6]. These carcinomas are formed in a stepwise process in which the cell progressively accumulates genetic aberrations until the normal cell cycle comes out of control and malignancy is initiated [73]. Further on, late genetic events will make the cell to completely lose its normal character and initiate an aggressive behavior [16,66].

From a prognostic point of view, our review demonstrates that DHGTC has the best prognosis, PDTC has an intermediate one, while ATC is characterized by a grim prognosis of just a few months. Even if the OS remains poor in ATC, some studies claim increased survival rates. Even though small patient lots were used; both Evans [26] and Lee [74] report sensibly longer survival rates in patients treated multimodally (e.g., immune-targeted therapies, radiotherapy, and surgery).

Clinical outcome data suggest that DHGTC has a close value of distant metastasis upon presentation to PDTC. Moreover, DHGTC has higher rates of extrathyroidal extension and lymph node involvement than PDTC, and interestingly enough has a much better prognosis. This might be due to the variated therapeutic variants available for the former [75]. As we noticed, the age upon initial diagnosis is higher for ATC than PDTC (*p* < 0.001) and ATC, respectively (*p* < 0.001). This could suggest that ATC needs a longer period of time to gather the mutational burden. Also, ATC prognosis worsens with increasing age [67,76].

Due to the limited therapeutic strategies, studies are now focusing on the molecular characterization of these aggressive carcinomas, in hope of finding new molecular targets or even new uses for existent therapies [77,78].

Clinical studies are currently evaluating multiple promising targeted therapies such as tyrosine kinase inhibitors, anti-BRAF, checkpoint inhibitors, and anti PPAR-G treatments [78]. Tiendje et al. [55] demonstrate in their significant cohort, that up to 33% of all ATC cases do harbor mutations that can be potentially inhibited by targeted therapy. Landa et al. [35] demonstrates with a big patient lot that ATC harbored PI3K mutations that make the cells susceptible to mTOR inhibitors, thus opening another potential therapy gate. Although BRAF-mutant ATC has an even worse prognosis, anti-BRAF drugs such as Dabrafenib in association with Trametinib greatly increased overall survival in patients [15,38,79,80]. Riccio et al. [81] prove with their meta-analysis that, by testing for this mutation a population of patients at higher risk for metastasis and higher mortality. They could benefit from focused surveillance and more aggressive treatments. Even though RAS itself cannot be targeted by treatments, downstream effectors in MAPK pathway can be intercepted. Selumetinib that proved to increase RAI uptake in refractory carcinomas [81,82]. TP53 mutation may lower the expression of sodium-iodine symporter and by that generate RAI refractoriness and worse prognostic [83]. TP53 targeted promising therapeutic mechanisms include protection of normal P53 and anti TP53 antibodies, none of which are currently available [84,85].

Nonetheless, given the fact that DHGTC emerged as a new entity in 2022, the literature behind it is obviously scarce. Our review was limited to four eligible studies on the mutational profile (468 patients) and 4 (227 patients) for the clinical and prognostic assessment, adding to a total of seven writings, one being common to both. This may lead, of course, to imprecise estimates concerning DHGTC and potentially limiting the generalizability to specific patient populations and statistical significance. Also, by limiting the language only to English, we may induce a risk of not covering the entire literature of interest.

Further studies on the subject should be carried on, mainly on DHGTC, as its characterization as a new clinicopathologic entity is continuously expanding, as well as retrospective studies using the 2022 WHO criteria should be carried on tissue archives. Bigger data sets would yield a better understanding of the clinical and molecular characterization of this particular malignancy.

## 5. Conclusions

This review collects clinical, prognostic and molecular data concerning the three aggressive types of thyroid carcinoma. Presented data suggests that DHGTC has a good prognosis, having a milder clinical evolution. PDTC has a moderate prognosis, in between DHGTC and PDTC. PDTC has by far the worst prognosis and it is characterized by frequent metastases, lymph node invasion and extrathyroidal extension. Mutationally, each cancer seems to have a particular molecular signature that may be the basis of new therapeutic possibilities.

## Data Availability

Data extracted from included studies is available upon reasonable request.

## Supplementary Materials

The following supporting information can be downloaded at: https://www.mdpi.com/article/10.3390/ijms26125535/s1.

## Abbreviations

AJCC	American Joint Committee for Cancer
ATC	Anaplastic thyroid carcinoma
DHGTC	Differentiated high-grade thyroid carcinoma
DTC	Differentiated thyroid carcinoma
FISH	Fluorescent in situ hybridization
NGS	New generation sequencing
OS	Overall Survival
PDTC	Poorly differentiated thyroid carcinoma
RAI	Radioactive iodine
TNM	Tumor node metastasis
WHO	World Health Organisation
