# Supplementary material for: Aggressive Thyroid Carcinomas Clinical and Molecular Features: A Systematic Review
Source: Int J Mol Sci. 2025 Jun 10;26(12):5535. doi: 10.3390/ijms26125535 (PMC12192656; doi:10.3390/ijms26125535)
Supplement: Supplementary file 1 [file ijms-26-05535-s001.zip › Supplemental File 1. JBI Critical Appraisal Checklist for Studies Reporting Prevalence Data.pdf]

JBI Critical Appraisal Checklist for Studies Reporting Prevalence Data				
Major Components	Response options			
Q1. Were the criteria for inclusion in the sample clearly defined?	Yes	No	Unclear	Not applicable (NA)
Q2. Were the study subjects and the setting described in detail?	Yes	No	Unclear	Not applicable (NA)
Q3. Was the exposure measured in a valid and reliable way?	Yes	No	Unclear	Not applicable (NA)
Q4. Were objective, standard criteria used for measurement of the condition?	Yes	No	Unclear	Not applicable (NA)
Q5. Were confounding factors identified?	Yes	No	Unclear	Not applicable (NA)
Q6. Were strategies to deal with confounding factors stated?	Yes	No	Unclear	Not applicable (NA)
Q7. Were the outcomes measured in a valid and reliable way?	Yes	No	Unclear	Not applicable (NA)
Q8. Was appropriate statistical analysis used?	Yes	No	Unclear	Not applicable (NA)

We used the JBI Critical Appraisal Checklist Tool to assess and report the risk of bias for every included study. We present below tables with results for all the included studies. Each positive (yes) answer was given one point. Studies scoring ≥ 5 were considered of good quality, whereas studies with scores of 4 were regarded as satisfactory quality.

Author, year	Q1	Q2	Q3	Q4	Q5	Q6	Q7	Q8	TOTAL Yes	Comment
Aslan et al. 2014 [23]	Yes	Yes	NA	Yes	NA	NA	Yes	Yes	5	Good
Bonhomme et al. 2017 [49]	Yes	Yes	NA	Yes	NA	NA	Yes	Yes	5	Good
Brignardello et al. 2014 [24]	Yes	Yes	Yes	Yes	NA	NA	Yes	Yes	6	Good
Duan et al. 2019 [25]	Yes	Yes	NA	Yes	NA	NA	Yes	Yes	5	Good
Evans et al. 2024 [26]	Yes	No	NA	Yes	Yes	Yes	Yes	Yes	6	Good
Fouchardiere et al. 2018 [27]	Yes	Yes	NA	Yes	No	No	Yes	Yes	5	Good
Glasser et al. 2016 [28]	Yes	No	NA	Yes	NA	NA	Yes	Yes	4	Satisfactory
Gu et al. 2024 [39]	Yes	No	NA	Yes	NA	NA	Yes	Yes	4	Satisfactory
Ibrahimpasic et al. 2014 [30]	Yes	Yes	NA	Yes	No	No	Yes	Yes	5	Good
Jeong et al. 2023 [31]	Yes	Yes	NA	Yes	NA	NA	Yes	Yes	5	Good
Jin et al. 2022 [32]	Yes	No	NA	Yes	NA	NA	Yes	Yes	4	Satisfactory
Kersting et al. 2021 [33]	Yes	Yes	Yes	Yes	NA	NA	Yes	Yes	6	Good
Kunte et al. 2022 [34]	Yes	Yes	NA	Yes	NA	NA	Yes	Yes	5	Good
Landa et al. 2016 [35]	Yes	NA	NA	Yes	NA	NA	Yes	Yes	4	Satisfactory
Latteyer et al. 2016 [50]	Yes	Yes	NA	Yes	NA	NA	Yes	Yes	5	Good
Panchangam et al. 2022 [14]	Yes	Yes	Yes	Yes	NA	NA	Yes	Yes	6	Good
Patil et al. 2025 [36]	Yes	Yes	Yes	Yes	NA	NA	Yes	Yes	6	Good
Paunovic et al. 2016 [37]	Yes	No	NA	Yes	NA	NA	Yes	Yes	4	Satisfactory
Pozdeyev et al. 2018 [51]	Yes	Yes	NA	Yes	NA	NA	Yes	Yes	5	Good
Ravi et al. 2019 [48]	Yes	No	NA	Yes	NA	NA	Yes	Yes	4	Satisfactory
Saito et al. 2024 [38]	Yes	Yes	NA	Yes	NA	NA	Yes	Yes	5	Good

Scholfield et al. 2025 [52]	Yes	Yes	NA	Yes	NA	NA	Yes	Yes	5	Good
Sherman et al. 2009 [39]	Yes	Yes	Yes	Yes	No	No	Yes	Yes	6	Good
Stenman et al. 2021 [53]	Yes	Yes	NA	Yes	NA	NA	Yes	Yes	5	Good
Swaak-Kragten et al. 2011 [40]	Yes	No	Yes	Yes	No	No	Yes	Yes	5	Good
Takano et al. 2007 [54]	Yes	Yes	NA	Yes	NA	NA	Yes	Yes	5	Good
Thompson et al. 2023 [41]	Yes	Yes	NA	Yes	NA	NA	Yes	Yes	5	Good
Tiedje et al. 2017 [55]	Yes	Yes	NA	Yes	NA	NA	Yes	Yes	5	Good
Toda et al. 2024 [56]	Yes	Yes	Yes	Yes	NA	NA	Yes	Yes	6	Good
Tondi Resta et al. 2024 [42]	Yes	Yes	NA	Yes	NA	NA	Yes	Yes	5	Good
Torous et al. 2024 [57]	Yes	Yes	NA	Yes	NA	NA	Yes	Yes	5	Good
Wendler et al. 2016 [43]	Yes	Yes	NA	Yes	NA	NA	Yes	Yes	5	Good
Wong et al. 2019 [44]	Yes	Yes	NA	Yes	NA	NA	Yes	Yes	5	Good
Wu et al. 2023 [45]	Yes	Yes	NA	Yes	Yes	NA	Yes	Yes	6	Good
Xu et al. 2022 [9]	Yes	Yes	NA	Yes	NA	NA	Yes	Yes	5	Good
Xu et al. 2023 [46]	Yes	Yes	NA	Yes	NA	NA	Yes	Yes	5	Good
Xu et al. 2022 [9]	Yes	Yes	NA	Yes	NA	NA	Yes	Yes	5	Good
Yamazaki et al. 2024 [58]	Yes	Yes	NA	Yes	NA	NA	Yes	Yes	5	Good
Yu et al. 2017 [47]	Yes	Yes	NA	Yes	NA	NA	Yes	Yes	5	Good
