# Supplementary material for: Aggressive Thyroid Carcinomas Clinical and Molecular Features: A Systematic Review
Source: Int J Mol Sci. 2025 Jun 10;26(12):5535. doi: 10.3390/ijms26125535 (PMC12192656; doi:10.3390/ijms26125535)
Supplement: Supplementary file 1 [file ijms-26-05535-s001.zip › Supplemental Table S1 - clinical.pdf]

Cancer type	Parameter	Weighted average	95% CI
DHGTC	Median age	54.8	[46.901; 62.132]
	Metastasis	0.238	[0.034; 0.516]
	ETE	0.635	[0.186, 0.732]
	Lymph node	0.431	[0.074; 0.755]
	Tumor size	4,810	[1.820; 7.533]
	Stage I	0.240	[0.008; 0.558]
	Stage II	0.301	[0.062; 0.553]
	Stage III	0.317	[0; 0.790]
	Stage IV	0.133	[0; 0.147]
PDTC	Median age	58,5	[55.363; 59.637]
	Metastasis	0.2117	[0.240; 0.386]
	ETE	0.5519	[0.414; 0.637]
	Lymph node	0.3222	[0.251; 0.456]
	Tumor size	4,89	[3.722; 7.020]
	Stage I	0.1392	[0.044; 0.214]
	Stage II	0.2015	[0.111; 0.251]
	Stage III	0.4553	[0.250; 0.500]
	Stage IV	0.2024	[0.175; 0.430]
	Overall survival	55,8	[21.537; 73.466]
ATC	Median age	69	[65.662; 70.246]
	Metastasis	0.4215	[0.373; 0.538]
	ETE	0.5851	[0.414; 0.811]
	Lymph node	0.4414	[0.339; 0.631]

	Tumor size	5.93	[4.109; 7.895]
	Stage IV-A	0.1181	[0.054; 0.155]
	Stage IV-B	0.4992	[0.366; 0.773]
	Stage IV-C	0.3827	[0.155; 0.527]
	6MO survival	0.5105	[0.188; 0.812]
	12MO survival	0.1906	[0.131; 0.311]
	24MO survival	0.1115	[0.054; 0.248]
	Overall survival	3.7	[4.093; 6.052]
