# Supplementary material for: Aggressive Thyroid Carcinomas Clinical and Molecular Features: A Systematic Review
Source: Int J Mol Sci. 2025 Jun 10;26(12):5535. doi: 10.3390/ijms26125535 (PMC12192656; doi:10.3390/ijms26125535)
Supplement: Supplementary file 1 [file ijms-26-05535-s001.zip › Supplemental Table S2 -mutations.pdf]

| Type of cancer | Gene alteration | Weighted average | 95% CI |
|---|---|---|---|
| **DHGTC** | B-RAF | 0.289 | [0.136; 0.524] |
| | All-RAS | 0.317 | [0; 0.566] |
| | TERT | 0.462 | [0.283; 0572] |
| | TP53 | 0.104 | [0; 0.507] |
| | PTEN | 0.100 | - (one value) |
| | PIK3CA | 0.030 | - (one value) |
| **PDTC** | B-RAF | 0.139 | [0.071; 0.239] |
| | All-RAS | 0.355 | [0.192; 0.425] |
| | TERT | 0.448 | [0.327; 0.516] |
| | TP53 | 0.169 | [0.048; 0.342] |
| | PTEN | 0.117 | [0; 0.183] |
| | PIK3CA | 0.052 | [0; 0.335] |
| **ATC** | B-RAF | 0.350 | [0.221; 0.417] |
| | All-RAS | 0.266 | [0.231; 0.298] |
| | TERT | 0.657 | [0.472; 0.703] |
| | TP53 | 0.614 | [0.457; 0.669] |
| | PTEN | 0.090 | [0.062; 0.206] |
| | PIK3CA | 0.170 | [0.103; 0.294] |

Supplemental Table S2. We present the mutations grouped by type of cancer, along with their computed weighted average and 95% CI for the individual values yielded by each article. DHGTC: differentiated high grade carcinoma; PDTC: poorly differentiated thyroid carcinoma; ATC: anaplastic thyroid carcinoma; 95% CI: 95% confidence interval.
